# Editorial: Promoting health and addressing disparities amongst Indigenous populations

**DOI:** 10.3389/fpubh.2024.1526515

**Published:** 2024-12-13

**Authors:** Esteban Ortiz-Prado, Rene L. Begay, Jorge Vasconez-Gonzalez, Juan S. Izquierdo-Condoy

**Affiliations:** ^1^One Health Research Group, Faculty of Health Science, Universidad de Las Americas, Quito, Ecuador; ^2^Centers for American Indian and Alaska Native Health, Colorado School of Public Health, University of Colorado Anschutz Medical Campus, Aurora, CO, United States

**Keywords:** Indigenous populations, health inequities, systemic discrimination, healthcare access, quality care

## Introduction

Indigenous populations worldwide endure persistent health inequities shaped by colonial legacies, systemic discrimination, and socio-economic marginalization. These disparities manifest in elevated rates of preventable diseases, limited access to healthcare, and poorer health outcomes compared to non-Indigenous populations. Discrimination can be a fundamental cause of the health inequalities that exist among Indigenous peoples. Additionally, it has a direct negative impact on health and wellbeing, as the mistreatment, stereotypes, and lack of quality care they experience discourage them from accessing health services. Therefore, generating multidimensional public policies that protect these groups is essential (1, 2). Despite global commitments to health equity, barriers such as cultural disconnects, geographic isolation, lack of resources, or geneal exclusion of Indigenous people, and inadequate policy implementation hinder meaningful progress. The studies in this editorial illuminate the multifaceted health challenges faced by Indigenous communities and underscore the urgent need for culturally grounded, community-driven solutions. By navigating through the breadth of research compiled here, we aim to highlight research studies that clearly advocate for Indigenous health equity and inspire action to bridge these gaps.

## Exploring the research and key findings

This editorial presents 19 papers exploring diverse health challenges and innovative interventions for Indigenous populations. These studies exemplify how integrating cultural knowledge and community engagement can drive impactful changes.

One of the central themes is access to healthcare. In Australia, the uptake of health assessments among Indigenous populations remains limited due to logistical barriers, cultural disconnects, and systemic inefficiencies (Usher et al.). Indigenous clinical leadership emerges as a crucial enabler, highlighting the importance of empowering communities to lead health initiatives. Similarly, in Mexico, the COVID-19 pandemic exacerbated reproductive health inequities, halving contraceptive use among Indigenous women (Castro-Porras et al.). These findings emphasize the fragility of healthcare systems during crises and the need for resilient, inclusive solutions.

Mental health is another recurring focus. The adaptation of mental health interventions for Diné adolescents during the pandemic underscores how culturally tailored approaches can mitigate the psychological impacts of isolation and systemic inequities (Allison-Burbank et al.). In parallel, the validation of historical loss scales for Native Hawaiian adults highlights the intergenerational trauma affecting mental health, pointing to the necessity of tools that capture culturally specific experiences (Antonio et al.).

Addressing maternal and child health disparities is also vital. In Ecuador, community-driven sexual education programs emerged as a promising strategy to reduce teenage pregnancies in Indigenous populations (Tituaña et al.). Similarly, research in Aboriginal and Torres Strait Islander communities in Australia revealed that culturally safe maternal care can help reduce stillbirth disparities (Massi et al.). For Diné toddlers, an innovative language intervention combined traditional values with developmental care to address delays caused by systemic neglect (Billey et al.). These studies collectively advocate for interventions that respect Indigenous cultural values while addressing health inequities.

The impact of systemic discrimination on Indigenous health is undeniable. Research on American Indian and Alaska Native populations documented pervasive experiences of exclusion, stigmatization, and physical and verbal threats can contribute to poorer health outcomes if not addressed by the larger population (Begay et al.). Similarly, colonial legacies have entrenched disparities in brain health, with a review calling for culturally aligned care models that prioritize community connections and resilience (Henderson et al.).

Finally, environmental health emerges as a critical domain. Water contamination caused by mining in Diné communities exemplifies how systemic injustices extend to resource access (Tsosie). Innovative solutions, such as point-of-use water filters, reflect the resilience and ingenuity of Indigenous communities in addressing these challenges.

## Summary of key contributions

- Preventative health assessments in indigenous populations of Australia (Usher et al.)

This study analyzed the uptake of health assessments by Indigenous Australians. Findings revealed barriers such as limited access to services, lengthy assessments, and cultural disconnects. Indigenous clinical leadership was identified as crucial to improving participation. The study emphasizes the need for culturally sensitive strategies to enhance health outcomes.

- Reduction in contraceptive use during COVID-19 in an Indigenous Mexican Community (Castro-Porras et al.)

This research documented a 50% decline in contraceptive use among Indigenous Mexican women during the pandemic. Challenges included supply disruptions and service inaccessibility, highlighting the need for resilient healthcare systems that address reproductive health needs during emergencies.

- Recommendations for Indigenous Substance Use Disorder (SUD) Treatment (Hirchak et al.)

A scoping review assessed effective interventions for Indigenous communities, emphasizing the integration of Indigenous knowledge and adapting Western frameworks. Community engagement and culturally centered approaches were highlighted as essential for improving treatment outcomes.

- Mapping the knowledge structure and trends in Australian Indigenous Health Research (Krahe et al.)

This scientometric analysis identified a shift from deficit-focused studies to applied, culturally safe approaches. The study calls for advancing equity through respectful collaborations with Indigenous communities and addressing structural barriers within healthcare systems.

- Colonial drivers and cultural protectors of brain health among Indigenous peoples (Henderson et al.)

This review explored how colonial legacies have shaped brain health disparities. It emphasized the need for culturally relevant care models and tools, as well as addressing systemic stressors like discrimination and unequal access to care.

- Small for gestational age and anthropometric body composition from early childhood to adulthood (Hansen et al.)

This cohort study examines changes in anthropometric measurements, including fat measures, across the life course comparing small for gestational age and non-small for gestational age individuals living in urban and remote communities.

- Honoring our teachings: children's storybooks as indigenous public health practice (Maudrie et al.)

This study described the development of a culturally grounded storybook promoting mental health and resilience among AIAN children. By integrating cultural teachings with public health guidance, the project highlighted storytelling as an effective community-based intervention.

- A community-embedded approach to increasing the health literacy of Aboriginal children in a regional area (Good et al.)

This study explores the implementation of a child-centered, co-designed and community-integrated program to improve health and wellbeing outcomes for Aboriginal children in the middle childhood years.

- A psychometric analysis of historical loss scales among native Hawaiian adults (Antonio et al.)

This study validated tools for measuring historical loss and its associated symptoms, such as depression and anxiety. It underscores the importance of addressing intergenerational trauma in developing culturally sensitive interventions for Native Hawaiian communities.

- **A** rural teledentistry care experience in a Mapuche Community in Chile (Beltrán et al.)

This study demonstrated the potential of teledentistry to address oral health disparities in rural Indigenous communities. The approach improved access to care and reduced health inequities for elders.

- Factors influencing survival and mortality in aboriginal Australians with bronchiectasis (Heraganahally et al.)

The study identified factors such as ICU visits and pseudomonas infections as increasing mortality risk, while higher BMI and better lung function were protective. These findings stress the need for targeted interventions to reduce mortality.

- Reducing teenage pregnancy in rural Ecuadorian Indigenous communities (Tituaña et al.)

The study emphasized the importance of culturally tailored sexual education programs to address teenage pregnancy. Community engagement and youth-focused approaches were deemed essential for reducing pregnancy rates among Indigenous adolescents.

- Adapting safety planning interventions for Diné communities (Allison-Burbank et al.)

This project culturally adapted mental health interventions for Diné adolescents during COVID-19. It highlighted the value of community-driven approaches in addressing mental health challenges.

- Development of “+language is medicine” for Navajo toddlers (Billey et al.)

The study created a culturally responsive intervention to address developmental delays in Diné toddlers. The program integrates Indigenous values and language, emphasizing culturally grounded approaches to early childhood health.

- Addressing cervical cancer disparities in Indigenous women in Latin America (Muslin)

This article highlighted structural and cultural barriers to HPV vaccination and cervical cancer screening. It advocates for community-driven, culturally tailored interventions to reduce disparities.

- Menopause, blood pressure, and osteoporosis in rural women (Jin et al.)

The study found associations between high blood pressure and osteoporosis in postmenopausal women, emphasizing the need for gender-sensitive healthcare strategies in resource-limited settings.

- Discrimination among American Indian and Alaska native populations (Begay et al.)

This study documented widespread experiences of discrimination and its detrimental health effects. It calls for public health strategies to mitigate these impacts on Indigenous communities.

- Mining legacies and water contamination in Navajo Communities (Tsosie)

This article explored water contamination challenges in Diné communities and proposed innovative, community-centered filtration solutions to improve water safety.

- Looking after Bubba for all our mob (Massi et al.)

This study addressed stillbirth disparities in Aboriginal communities, advocating for culturally safe, family-centered approaches and Indigenous birthing practices to improve maternal health outcomes.

## Conclusion

The collective insights from these 19 studies underscore a compelling call to action: addressing health disparities among Indigenous populations is not only a moral imperative but a vital step toward achieving global health equity. Promoting Indigenous health demands more than addressing systemic inequities—it requires valuing cultural knowledge, empowering community leadership, and prioritizing co-designed, culturally sensitive solutions. The articles in this Research Topic provide valuable insights into the unique health challenges faced by Indigenous communities and offer pathways to bridge these gaps. However, much work remains to be done. Centering Indigenous voices in research, policy, and practice is essential for driving meaningful and sustainable progress. Only through sustained efforts and equity-driven policies can we hope to close the health gap and secure a healthier, more just future for all Indigenous populations.
